# Clinical, radiological and therapeutic features of keratocystic odontogenic tumours: a study over a decade

**DOI:** 10.4317/jced.51408

**Published:** 2014-07-01

**Authors:** Rocío Sánchez-Burgos, Javier González-Martín-Moro, Elia Pérez-Fernández, Miguel Burgueño-García

**Affiliations:** 1 Oral and Maxillofacial Department, Hospital Universitario de Canarias, Tenerife, Spain; 2 Oral and Maxillofacial Department, Hospital Universitario La Paz, Madrid, Spain; 3 Biostatistics Unit, IdiPAZ, Hospital Universitario La Paz, Madrid, Spain

## Abstract

Factors associated with the potential for recurrence of keratocystic odontogenic tumours (KCOT) still remain to be clearly determined and no consensus exists concerning the management of KCOT. The purpose of this study was to evaluate different clinical factors associated with KCOT and its treatment methods. A retrospective review was performed of 55 cases treated from 2001 to 2010. Of the 55 cases, 27% were associated with an impacted or semi-impacted tooth. The majority of the lesions (82%) were located in tooth-bearing areas, and the overall mandibular to maxilla ratio of tumour occurrence was 5:1. The treatment options included enucleation, marsupialisation, or peripheral ostectomy, with or without the use of Carnoy´s solution. Recurrence was found in 14 cases (25%). No significant association was seen between recurrence and age, symptomatic cases, location of the lesion, or unilocular or multilocular appearance. The recurrence rate was higher in the group with tooth involvement, more marked in cases with third molar involvement. Statistical analysis showed a significant relation between recurrence and the type of treatment, with higher rates in cases treated with enucleation associated with tooth extraction. In our series, those cases with a closer relation with dental tissues showed a higher risk of recurrence, suggesting the need for a distinct classification for peripheral variants of KCOT.

** Key words:**Keratocystic odontogenic tumour, Odontogenic keratocyst, Odontogenic cysts, Keratocyst, Carnoy’s solution.

## Introduction

An odontogenic keratocyst [OKC] is an epithelial cyst lesion commonly located in the maxilla and mandible with a significant potential for growth, expansion and local invasion. The first description of an OKC was by Philipsen in 1956. OKCs account for 12-14% of all odontogenic cysts of the jaws, with a predilection for the posterior region of the mandible. Histologically, a fibrous capsule and a lining of keratinized squamous epithelium characterize OKCs. Recurrence has been described up to 10 years after treatment, though it is more common during the first 5-7 years. The potential risk of recurrence and the long intervals reported explain the need for long-term follow-up.

Based on the biological behaviour of OKCs and recent research about chromosomal and genetic abnormalities, in 2005 the WHO [World Health Organization] Working Group considered the OKC to be a benign tumour and recommended the term keratocystic odontogenic tumour [KCOT] (1). KCOT is distinct from the orthokeratini-zing odontogenic cyst, which is considered an odontogenic cyst.

No consensus exists concerning the management of KCOT. Conservative treatment includes enucleation, curettage or marsupialization. More aggressive management is based on ostectomy, en bloc resection or use of chemical agents like Carnoy’s solution. The goal of surgical treatment is to control potential recurrence with the least morbidity. However, no good evidence is currently available regarding the best treatment option (2).

We report a retrospective study of all KCOTs treated over a period of 10 years at the La Paz University Hospital, Madrid, with statistical analyses of epidemiological and clinical characteristics, management and course, and discuss possible factors related to recurrence.

## Material and Methods

A retrospective analysis was performed of all 55 cases of KCOTs treated at La Paz University Hospital between 2001 and 2010. Patients were included if they had been treated surgically at this centre during the study period and the lesion was diagnosed histologically as KCOTs with parakeratosis. Patients were excluded if they had basal cell nevus syndrome, a histopathological diagnosis of an orthokeratinized variant, or if they had been followed-up for less than one year.

Demographic data collected included age and gender. The study population was divided into two groups, according to the clinical and diagnostic data. The first group comprised symptomatic cases with clinical findings like pain, mandibular nerve dysaesthesia, swelling or suppuration. The second group included asymptomatic cases, with the diagnosis made incidentally by radiographic findings or during the course of routine buccal examination.

The location of the cyst was divided into those in the maxilla, either in anterior [incisor and canine] or posterior [premolar and molar] regions; and those in the mandible. The mandible itself was divided into different anatomic areas: symphysis, parasymphysis, body, angle, ramus, condyle, and coronoid process.

Radiographic findings described included single or multiple location, rhizolysis, and radicular displacement. Information was also recorded about whether a tooth was related with the lesion, and if so, the type of relation: coronal, periapical or parafollicular location. Histological findings were also recorded.

The treatment options described included enucleation, marsupialisation, or peripheral ostectomy, with or without the use of Carnoy´s solution. Enucleation included enucleation of the cyst with curettage and/or peripheral ostectomy in all cases. In cases with dental involvement, the management included tooth extraction or apicectomy in an endodontically treated tooth. The use of Carnoy’s solution could be combined with any other treatment. Ostectomy was defined as block resection of the cyst with marginal healthy bone. Any reconstructive technique used was also recorded, such as the use of autologous bone or any heterologous material.

The follow-up period was recorded, and any complication during the treatment or the follow-up was noted. Data were also recorded on the incidence of recurrence, as well as the time of recurrence, localization and management. The time of recurrence was calculated as the number of months between the surgical treatment and the biopsy of the recurrence. Surgical management of the recurrence was classified in the same groups as for treatment of the primary lesions. Note was also made of any reconstructive technique employed.

The statistical analysis was done with SPSS for Windows [Release 9.0] to search for associations between variables, including age, gender, location, symptoms, radiological appearance, tooth involved, treatment modality and recurrence. Qualitative data are described by absolute and relative frequencies. Quantitative data are described by mean and standard deviation or the median and interquartile range [p25-p75]. Univariate associations were studied with a non-parametric test because of the small sample size. Quantitative data were tested by the Mann-Whitney rank-sum test and qualitative data by Fisher’s exact test. Two-sided tests were used and a P value less than 0.05 was considered statistically significant.

## Results

**Figure 1:**
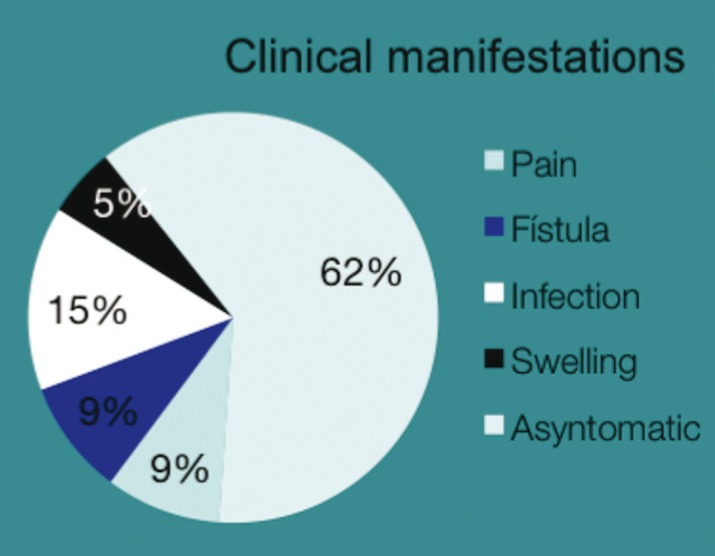
Distribution of clinical manifestations.

The study included a total of 55 patients, with a slight male predominance [56%]. The mean age was 42 years, with peaks in the second and fifth decades. Clinically, though 62% of the lesions were diagnosed incidentally during routine dental examination, 38% produced symptoms including swelling, pain and discharge (Fig. 1). No significant associations were found between symptomatic cases and unilocular or multilocular lesions or between asymptomatic cases and unilocular or multilocular lesions. Nor were significant relations found between symptomatic or asymptomatic cases and single or multiple anatomical locations.

Radiologically, 71% of the tumours were identified as unilocular lesions whereas 29% had a multilocular appearance. Of the 55 cases, 15 [27%] were associated with an impacted or semi-impacted tooth. The majority of the lesions [82%] were located in tooth-bearing areas, most commonly in a periapical zone [74%]. Only one case caused root resorption and two cases made adjacent tooth roots divergent.

The overall mandibular to maxilla ratio of tumour occurrence was 5:1, with 81% of the lesions located in the mandible, most frequently in the body [20%], angle [18%] and vertical ramus [10%]. In 30% of the mandibular cases the tumour involved more than one anatomical area, most frequently affecting the body, angle and vertical ramus [10%]. Only 16% of the lesions occurred in the maxilla, most in the posterior region [13%].

The most frequent surgical treatment was enucleation associated with peripheral ostectomy or curettage, in 47% of the cases. In 30% of the patients, this treatment was associated with extraction of the associated tooth. In 11% of cases, the lesions were treated with enucleation and apicectomy of the associated tooth. Enucleation in combination with Carnoy’s solution, segmental mandibulectomy, and marsupialization were each used in 4% of cases.

**Figure 2:**
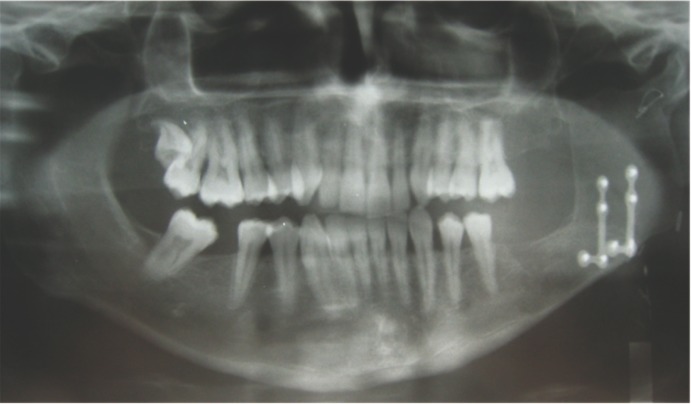
Left mandibular ramus defect recontructed by iliac microvascular flap.

In 7 cases autologous bone was used to reconstruct bone defects; in 4 cases with an iliac crest bone graft, and in 1 case with a combination of iliac crest bone and calvarian bone. In one patient a microvascularized fibula flap was employed to reconstruct an important tumour defect and in the other case a microvascularized iliac crest flap was used for the same purpose (Fig. 2).

**Figure 3:**
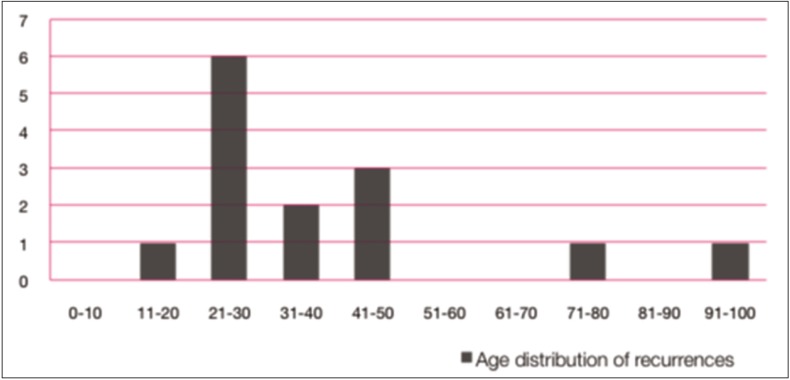
Age distribution of recurrences.

**Figure 4:**
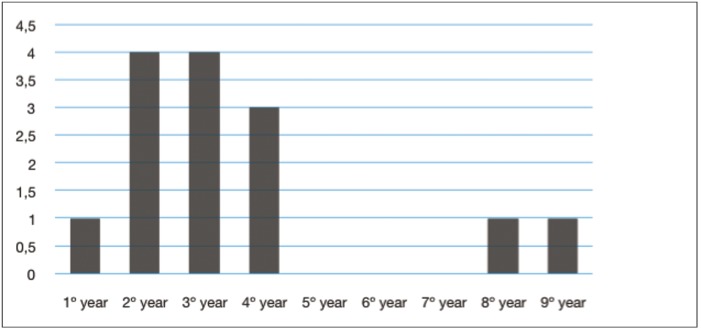
Follow-up distribution of recurrences: the recurrences were usually diagnosed during the first 5 years postoperatively.

The average follow-up period was 5 years. Recurrence was found in 14 cases [25%]. The most frequent location of recurrence was the mandibular body [7/14] and ramus [4/14]. The age distribution at recurrence was similar to the primary cases, showing a peak in the second decade (Fig. 3). All the recurrences were asymptomatic at the time of diagnosis except for 2 cases, one with inflammation and the other with dysaesthesia of the mandibular nerve. The recurrences were usually diagnosed during the first 5 years postoperatively, with a mean disease-free interval of 38 months [range: 8 months to 9 years] (Fig. 4). Treatment of recurrence included enucleation with curettage or peripheral ostectomy [8/14], associated with tooth extraction [4/14] and Carnoy’s solution [2/14]. Only one patient had multiple recurrences.

The statistical analysis showed no association between age and recurrence, although there was a higher rate of recurrence in men [31%] than women [17%]. The mean age at the time of first intervention in the recurrence group was slightly lower than in the non-recurrence group [37 years vs. 43 years, respectively], with no significant difference. Nor was a significant relation found between symptomatic cases and recurrence, though the rate of recurrence was higher than in the asymptomatic cases [31% vs. 21%, respectively].

No significant association was seen between recurrence and anatomical location of the lesion, although KCOTs located in the mandible showed a higher recurrence rate than those in the maxilla [29% vs. 11%, respectively]. Furthermore, 35% of the lesions involving more than one anatomical area developed a recurrence [6/17]. Half of those cases that extended into the body, angle or ramus experienced a recurrence [3/6]. Nevertheless, statistical analysis showed no significant difference in recurrence between those lesions with multiple anatomical locations and those with a single anatomical location. Concerning the anatomical area, lesions involving the mandibular ramus showed a higher recurrence rate [40%] than those involving other mandibular areas [17%], but with no significant difference. Nor was there a significant association between unilocular or multilocular lesions and recurrence, with a similar recurrence rate in both groups.

In cases associated with one or more teeth, the recurrence rate was 40% whereas in the group with no tooth involvement the recurrence rate was 20%. The recurrence rate was higher in cases with third molar involvement [40%] than those without third molar involvement [17%]. In cases with other molar involvement the recurrence rate was 20% compared with 15% in the group without molar involvement, although these associations were not significant.

**Figure 5:**
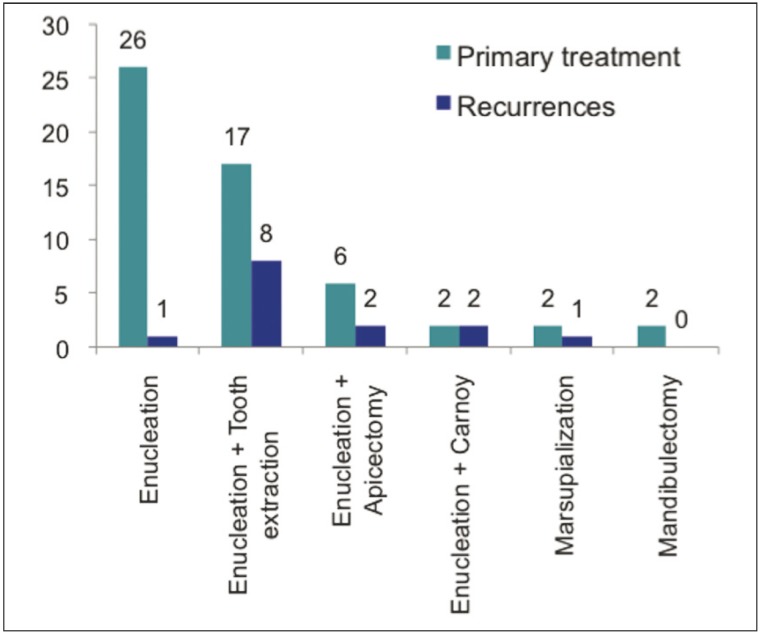
Distribution of recurrences in relation to primary treatment: patients who had recurrences were significantly more likely to have been treated with enucleation associated with tooth extraction.

**Figure 6:**
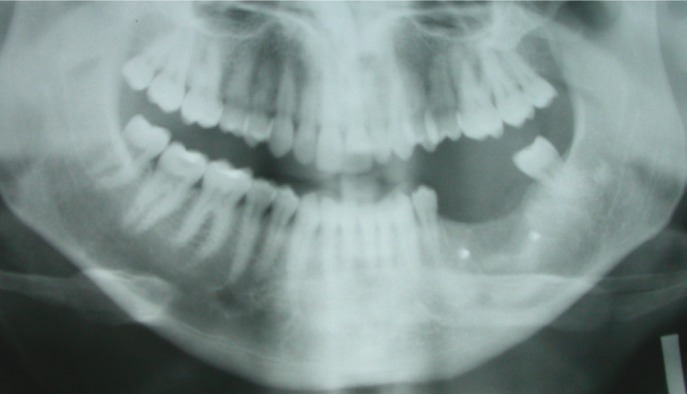
Recurrence located in an iliac crest bone graft on the left mandible molar area.

Concerning the surgical treatment employed, enucleation and curettage or peripheral ostectomy were associated with the lowest rate of recurrence [3.8%]. The recurrence rate in cases treated by enucleation and tooth extrac-tion was relatively high [43.5%]. Two of the six patients treated with enucleation and apicectomy experienced a recurrence, as did both patients treated with enucleation and Carnoy’s solution. One of the two patients treated with marsupialization also experienced a recurrence. There were no recurrences in the group treated by mandibulectomy (Fig. 5). One case of recurrence took place in an iliac crest bone graft (Fig. 6). Statistical analysis showed a significant association between recurrence and the type of treatment. Patients who had recurrences were significantly more likely to have been treated with enucleation associated with tooth extraction or apicectomy than patients who were treated with enucleation alone [P=0.001]. Cases treated under general anaesthesia experienced a recurrence rate of 33%, which differed significantly [P=0.026] compared with cases treated under local anaesthesia, who experienced no recurrences.

## Discussion

Our findings of a mean age of 42 years and the male predominance are similar to those of other studies (3,4). Clinically, 38% of our patients were symptomatic at the time of diagnosis. This implies that those KCOTs that present with symptoms of inflammation may have large areas with inflammatory cells. This implication has to be taken into account because of the high risk of a false-negative result from an aspiration or incisional biopsy. Radiologically, only 28% of the lesions showed the typical radiographic features, such as a multilocular appearance. The only reliable radiographic parameter described is the lack of cortical expansion in most KCOTs compared with odontogenic cysts or ameloblastomas, tending to hollow the mandible and fenestrate the lingual cortex (5).

Our results concerning the location are also consistent with the findings of other reports (3,4), with the most common location being the body, angle and mandibular ramus, and most lesions located in the tooth-bearing periapical areas. This could be explained by the hypothesis that KCOTs originate from remnants of the dental lamina or the basal layer of oral epithelium (6). Possible offshoots of the dental lamina could be located distal to the third molar and these epithelial residues may be related to the formation of a KCOT. However, there is little evidence to support this hypothesis.

In some KCOTs the connective tissue contains islands of epithelium or separate daughter cysts, often found where the wall of the cyst is attached to the mucosa. Most of these perforations are found high at the ascending ramus on top of the third molar and close to the bone, not in contact with dental lamina (7). Lesions penetrating soft tissues in areas difficult to handle like the mandibular ramus could explain recurrences in these cases; indeed, the mandibular ramus was the second most frequent location of recurrence in our series. Based on this theory, some authors have proposed the excision of an area of mucosa where the cyst is attached (5). We also had a case of recurrence in an iliac crest bone graft, as has been reported elsewhere; this may originate from outside the bone graft, probably from the soft tissues covering it (8).

The overall rate of recurrence in our series was 25% after a mean follow-up period of 5 years. Recurrence rates reported range widely, from 5% to 70% (9,10). No significant association was found between sex or age and recurrence, though some authors have reported higher recurrence rates in younger patients (11) as well as in patients in the fifth decade of life (4).

Though the recurrence rate did not correlate with cyst size in other reports (12), one third of our cases of multiple anatomical extension showed recurrence and half of the cases extending into the body, angle and ramus recurred. However, statistical analysis showed no significant association between recurrence and a multiple or single location. The significant association between recurrence and cases treated under general anaesthesia suggests a greater likelihood of recurrence of more complex lesions, which require general anaesthesia for surgical management.

Daughter cyst formation has also been associated with high recurrence rates, though this has not been accepted unanimously. The use of chemical curettage (13), cryotherapy (14,15), or resection without continuity (16) has been advocated to ensure removing remaining epithelium. However, in our series, both the cases treated with enucleation in combination with Carnoy’s solution developed a recurrence. This may have been because of the ability of Carnoy’s solution to fix a daughter cyst located in bone but not in gingival tissues.

The cases treated with enucleation and curettage or peripheral ostectomy, considered by many authors to be the minimal treatment required (17,18), showed a significantly lower rate of recurrence than the cases treated with the same treatment associated with tooth apicectomy or extraction. The fact that a tooth was involved in the cyst appears to be an important risk factor for the development of recurrence. Furthermore, the cases with at least one tooth involved showed a 20% higher tendency to experience recurrence compared with the cases with no relation with a tooth. Concerning the particular tooth, third molar involvement was associated with a higher rate of recurrence [40%] compared with other teeth [17%]. Chirapatholmsakul et al. reported that most recurrent lesions were found in the symphysis-body region because the tendency is to treat this region more conservatively (19). In our series, however, all the lesions with tooth involvement were treated with tooth extraction or apicectomy and this treatment appears to be related with recurrence. The higher recurrence rate in these cases may be explained by the closer relation of the cyst with the dental, periodontal and gingival tissues, which leads to the need for extraction of the tooth involved. This situation may involve incomplete removal of the KCOT due to adherence of the thin lining wall of the tumour to adjacent soft tissues in dentate areas. This is consistent with the theory mentioned earlier of the origin of these lesions from the oral mucosa and then their extension into the bone (5,7).

## Conclusions

In our series, those cases with a closer relation with dental tissues showed a higher risk of recurrence. In fact, a future question to be examined concerns the need for a distinct classification for peripheral variants of KCOT, as by definition the WHO included the intraosseus parakeratinised variant under the nomenclature of KCOT. This is supported by the requirement for a different treatment protocol for peripheral and intraosseus variants of KCOT (20).
